# Identifying RNA N^6^-Methyladenosine Sites in *Escherichia coli* Genome

**DOI:** 10.3389/fmicb.2018.00955

**Published:** 2018-05-14

**Authors:** Jidong Zhang, Pengmian Feng, Hao Lin, Wei Chen

**Affiliations:** ^1^Department of Immunology, Zunyi Medical College, Zunyi, China; ^2^Hebei Province Key Laboratory of Occupational Health and Safety for Coal Industry, School of Public Health, North China University of Science and Technology, Tangshan, China; ^3^Key Laboratory for Neuro-Information of Ministry of Education, Center for Informational Biology, School of Life Science and Technology, University of Electronic Science and Technology of China, Chengdu, China; ^4^Department of Physics, Center for Genomics and Computational Biology, School of Sciences, North China University of Science and Technology, Tangshan, China

**Keywords:** N^6^-methyladenosine, machine learning method, nucleotide physicochemical properties, microbial genome, pseudo nucleotide composition

## Abstract

N^6^-methyladenosine (m^6^A) plays important roles in a branch of biological and physiological processes. Accurate identification of m^6^A sites is especially helpful for understanding their biological functions. Since the wet-lab techniques are still expensive and time-consuming, it's urgent to develop computational methods to identify m^6^A sites from primary RNA sequences. Although there are some computational methods for identifying m^6^A sites, no methods whatsoever are available for detecting m^6^A sites in microbial genomes. In this study, we developed a computational method for identifying m^6^A sites in *Escherichia coli* genome. The accuracies obtained by the proposed method are >90% in both 10-fold cross-validation test and independent dataset test, indicating that the proposed method holds the high potential to become a useful tool for the identification of m^6^A sites in microbial genomes.

## Introduction

At present, ~150 kinds of RNA modifications have been found in different RNA species (Boccaletto et al., 2018), which not only enrich the genetic information, but also play critical roles in a variety of biological processes as mentioned in a recent review (Roundtree et al., 2017). Among these modifications, the N^6^-methyladenosine (m^6^A) is the most abundant posttranscriptional modification and has been found in the three domains of life. m^6^A has been found to participate in various biological activities, such as mRNA splicing (Nilsen, 2014), mRNA translation (Wang et al., 2015), mRNA maturation (Hoernes et al., 2016), stem cell proliferation (Bertero et al., 2018), and even a series of diseases (Zhang et al., 2016; Cui et al., 2017; Li et al., 2017).

In order to reveal its biological functions, different kinds of high-throughput sequencing techniques have been proposed to map the locations of m^6^A on genome wide (Dominissini et al., 2013; Linder et al., 2015; Wan et al., 2015; Hong et al., 2018). Although these techniques promoted the research progress on understanding the biological functions and the identification of RNA modifications, they are still labor-intensive and cost-ineffective. In addition, the resolution of detecting m^6^A sites for most techniques is still not satisfactory. Therefore, it's necessary to develop novel methods to detect m^6^A sites.

Giving the credit to the experimental data yielded by these high-throughput sequencing techniques as reported in a recent work (Chen X. et al., 2017), some machine learning based computational methods have been proposed to identify m^6^A sites (Chen et al., 2015a,b, 2016a, 2017b,c; Zhou et al., 2016). Although these methods are really good complements to experimental methods for detecting m^6^A sites, to the best of our knowledge, so far there is no computational tool available for detecting m^6^A sites in microbial genomes.

Stimulated by the successful applications of machine learning methods in computational genomics and proteomics (Chen et al., 2012; Feng et al., 2013; Cao et al., 2016, 2017a,b; Hu et al., 2018), in the present work, we presented a support vector machine (SVM) based method for identifying m^6^A sites in the *Escherichia coli* (*E. coli*) genome. By encoding the RNA sequences using nucleotide chemical property and accumulated nucleotide frequency, the proposed method obtained promising performances in 10-fold cross validation test. Moreover, we also validated the method on the independent dataset and obtained satisfactory results.

## Materials and methods

### Benchmark dataset

The m^6^A site containing sequences of *E. coli* genome were obtained from the RMBase database (Xuan et al., 2018). All the sequences are 41 bp long with the m^6^A site in the center. To overcome redundancy and reduce the homology bias, sequences with more than 80% sequence similarity were removed by using the CD-HIT program (Fu et al., 2012). After such a screening procedure, 2,055 m^6^A site containing sequences were retained and regarded as positive samples.

The negative samples (non-m^6^A site containing sequences) were obtained by choosing the 41-bp long sequences with the central adenosine that was not experimentally confirmed occurring methylation on its 6th nitrogen. By doing so, we could obtain a large number of negative samples. After removing sequences with identify >80%, the number of negative samples are still dramatically larger than that of positive samples. To balance out the numbers between positive and negative samples in model training, we randomly picked out the same number of negative samples and repeated this process 10 times. Therefore, 10 negative subsets were obtained, and each of them includes 2,055 non-m^6^A site containing sequences. The positive and negative samples thus obtained are provided in Supplementary Material.

### Sequence encoding scheme

Inspired by recent studies (Chen et al., 2016b,c,d, 2017a,d; Feng et al., 2017), in order to transfer the RNA sequences into discrete vectors that can be recognized and handled by machine learning methods, we encoded RNA sequences using nucleotide chemical properties and accumulated nucleotide frequency. Their brief descriptions are as following.

The four nucleotides, namely, adenine (A), guanine (G), cytosine (C), and uracil (U) can be classified into three different groups according to their physicochemical properties, i.e., ring structures, secondary structures, and chemical functionality (Chen et al., 2016b,c,d, 2017a,d; Feng et al., 2017). Therefore, based on the different physicochemical properties, the four coordinates (1, 1, 1), (0, 0, 1), (1, 0, 0), and (0, 1, 0) were used to represent the four bases (A, C, G, and U) of RNA, respectively.

In order to include nucleotide composition surrounding the modification site as well, the accumulated nucleotide frequency of any nucleotide *n*_*j*_ at position *i* was also used to represent RNA sequences and was defined as

(1) $$d_{i}=\frac{1}{\left|N_{i}\right|}\sum_{j=1}^{l}f(n_{j}),\;f\left(n_{j}\right)=\{\begin{array}{l} 1\;if\;n_{j}=q\\ 0\;other\;cases \end{array}$$

where |*N*_*i*_| is the length of the sliding substring concerned, *l* denotes each of the site locations counted in the substring, *q*ϵ{A, C, G, U}.

By integrating both nucleotide physicochemical properties and accumulated nucleotide frequency, an *L nt* long RNA sequence could be represented a 4*L*-dimensional vector (Chen et al., 2016b,c,d, 2017a,d; Feng et al., 2017).

### Support vector machine

As an efficient supervised machine learning algorithm, SVM has been widely used in the realm of bioinformatics (Cao et al., 2014; Li et al., 2017; Wang et al., 2017b; Zhang et al., 2017). Its basic idea is to transform the input data into a high dimensional feature space and then determine the optimal separating hyperplane.

In the current study, the implementation of SVM was performed by using the LibSVM package 3.18, available at http://www.csie.ntu.edu.tw/~cjlin/libsvm/. The radial basis kernel function (RBF) was used to obtain the classification hyperplane. The grid search method was applied to optimize its regularization parameter *C* and kernel parameter γ.

### Evaluation metrics

The performance was evaluated by using the following four metrics, namely sensitivity (*Sn*), specificity (*Sp*), Accuracy (*Acc*), and the Mathew's correlation coefficient (*MCC*), which can be expressed as

(2) $$\{\begin{array}{l} Sn=\frac{TP}{TP+FN}\times 100\%\\ Sp=\frac{TN}{TN+FP}\times 100\%\\ Acc=\frac{TP+TN}{TP+FN+TN+FP}\times 100\%\\ MCC=\frac{\left(TP\times TN\right)-\left(FP\times FN\right)}{\sqrt{\left(TP+FN\right)\times \left(TP+FP\right)\times \left(TN+FN\right)\times \left(TN+FP\right)}} \end{array}$$

where *TP, TN, FP*, and *FN* represent true positive, true negative, false positive, and false negative, respectively.

To further evaluate the performance of the current method more objectively, inspired by recent works (Wang et al., 2017a), the ROC (receiver operating characteristic) curve was also plotted. Its vertical coordinate indicates the true positive rate (sensitivity) and the horizontal coordinate indicates the false positive rate (1-specificity). The area under the ROC curve (auROC) is an indicator of the performance quality of a binary classifier, i.e., the value 0.5 of auROC is equivalent to random prediction while the value 1 of auROC represents a perfect one.

## Results and discussions

### Performance for m^6^A site identification

In statistical prediction, independent dataset test, *K*-fold cross-validation test and jackknife test are often used to derive the metric values for a predictor (Chou, 2011). In order to saving computational time, the 10-fold cross-validation test was used to examine the performance of the proposed method. In 10-fold cross-validation test, the samples in the dataset are randomly partitioned into 10 equal sized sub-datasets. Of the 10 sub-datasets, a single sub-dataset is retained as the validation data for testing the model, and the remaining 9 sub-datasets are used as training data. The process is then repeated 10 times, with each of the 10 sub-datasets used exactly once as the validation data.

By encoding RNA sequences using nucleotide chemical property and accumulated nucleotide frequency, each sample in the dataset was represented by a (4 × 41) = 164-dimensional vector and used as the input of SVM. The 10-fold cross-validation test results for identifying m^6^A sites in *E. coli* were listed in Table 1. In addition, to demonstrate that whether its accuracy is sensitive to the selection of negative data, the method was also tested on the other nine negative datasets, respectively. Their predictive results of the 10-fold cross-validation were also provided in Table 1.

**Table 1:** The 10-fold cross validation predictive results by using different negative datasets for identifying m^6^A sites in *E. coli*.

**Dataset**	**Sn (%)**	**Sp (%)**	**Acc (%)**	**MCC**
Negative set 1	100.00	98.59	99.29	0.98
Negative set 2	100.00	98.78	99.39	0.98
Negative set 3	100.00	98.44	99.22	0.98
Negative set 4	100.00	98.88	99.44	0.98
Negative set 5	100.00	98.44	99.22	0.98
Negative set 6	100.00	98.49	99.25	0.98
Negative set 7	100.00	98.54	99.27	0.98
Negative set 8	100.00	98.69	99.34	0.98
Negative set 9	100.00	98.49	99.25	0.98
Negative set 10	100.00	98.25	99.12	0.97
Average	100.00	98.56	99.28	0.98

**Figure 1:**
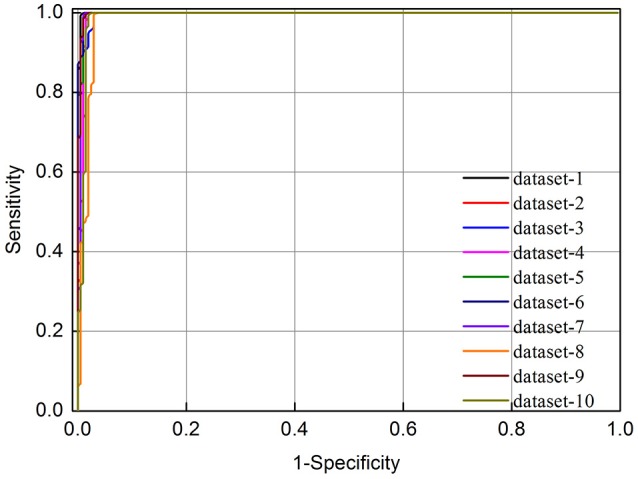
The ROC curves of 10-fold cross validation test for identifying m^6^A sites in *E. coli* based on different negative datasets. The vertical coordinate is the true positive rate (*Sn*) while horizontal coordinate is the false positive rate (1-*Sp*).

As indicated in Table 1, we found that the predictive accuracy is not affected by the selection of negative data. In addition, the 10 ROC curves obtained based on the 10 different negative datasets were also plotted in Figure 1. It was found that their auROCs are all higher than 0.98. These results demonstrate the reliability and robustness of the model developed in this study.

### Comparison with other methods

In order to demonstrate the effectiveness of nucleotide chemical property and accumulated nucleotide frequency for identifying m^6^A sites in *E. coli*, we compared the performance of the proposed method with that of the method based on other commonly used RNA sequence features. Chen et al. have proposed the pseudo nucleotide composition (PseKNC) to represent RNA sequences (Chen et al., 2014a,b), in which both the local and global sequence order information w included. Since it has been proposed in 2014, PseKNC have been used in in many branches of computational genomics (Guo et al., 2014; Lin et al., 2014, 2017). Therefore, we employed the SVM to perform the comparisons between the model based on nucleotide chemical property and accumulated nucleotide frequency features and that based on the PseKNC features (Chen et al., 2015a). The 10-fold cross-validation test results were listed in Table 2.

**Table 2:** Comparison of different parameters for identifying m^6^A sites in *E. coli*.

**Parameters**	**Sn (%)**	**Sp (%)**	**Acc (%)**	**MCC**
PseKNC	65.74	60.29	63.02	0.26
Secondary structure	67.06	60.73	63.89	0.28
Our method	100.00	98.56	99.28	0.98

As indicated in a recent study (Schwartz et al., 2013), the m^6^A modification is also affected by RNA secondary structures. Therefore, we performed the prediction of m^6^A sites by using RNA secondary structure. To this end, all the sequences in the benchmark dataset were encoded by using their second structures. The details about the encoding scheme based on secondary structures can be found in a recent work (Xue et al., 2005). By doing so, each RNA sequence is converted to a 32 dimensional vector (Xue et al., 2005) and used as the input feature of SVM. Its 10-fold cross-validation test results were also listed in Table 2.

As shown in Table 2, the predictive performance of the method based on nucleotide chemical property and accumulated nucleotide frequency is dramatically higher than that based on PseKNC and RNA secondary structure.

### Validation on independent dataset

The proposed method trained based on the benchmark dataset from the *E. coli* genome was further used to identify the m^6^A sites in the *P. aeruginosa* genome. For this purpose, we firstly collected the 5,814 experimentally confirmed m^6^A sites from the RMBase to form an independent dataset, which is given in Supporting Information S2. Of the 5,814 m^6^A sites in the *P. aeruginosa*, 5,809 were correctly identified, indicating that the proposed method is really quite promising for identifying m^6^A sites in microbial genomes.

## Conclusion

In this study, we present a computational method to identify m^6^A sites in the *E. coli* genome by encoding the RNA sequences using nucleotide chemical property and accumulated nucleotide frequency. The results obtained based on the benchmark dataset and independent dataset demonstrate that the proposed method is powerful and promising in discovering m^6^A sites. We hope that the proposed method will be helpful for the future research on m^6^A sites in microbial genomes.

Since user-friendly and publicly accessible web-servers (Feng et al., 2018)and databases (Liang et al., 2017) represent the direction of developing new prediction method, we will make efforts in our future work to provide a web-server for the method presented in this paper.

## Author contributions

HL and WC: conceived and designed the experiments; JZ and PF: performed the experiments; HL and WC: wrote the paper.

### Conflict of interest statement

The authors declare that the research was conducted in the absence of any commercial or financial relationships that could be construed as a potential conflict of interest.
